# Impact of the COVID-19 pandemic on psychological well-being of students in an Italian university: a web-based cross-sectional survey

**DOI:** 10.1186/s12992-021-00680-w

**Published:** 2021-04-06

**Authors:** Leonardo Villani, Roberta Pastorino, Enrico Molinari, Franco Anelli, Walter Ricciardi, Guendalina Graffigna, Stefania Boccia

**Affiliations:** 1 Section of Hygiene, University Department of Life Sciences and Public Health —Università Cattolica del Sacro Cuore, Rome, Italy; 2 Department of Woman and Child Health and Public Health - Public Health Area, Fondazione Policlinico Universitario A. Gemelli IRCCS, Rome, Italy; 3 Dipartimento di Psicologia, Università Cattolica del Sacro Cuore, Milan, Italy; 4 EngageMinds HUB – Consumer, Food & Health Engagement Research Center, Università Cattolica del Sacro Cuore, Cremona, Italy; 5 Università Cattolica del Sacro Cuore, Milan, Italy

**Keywords:** COVID-19, Pandemics, Mental health, Anxiety, Depression, Students

## Abstract

**Background:**

Italy was the first European country to implement a national lockdown because of the COVID-19 pandemic. Worldwide, this pandemic had a huge impact on the mental health of people in many countries causing similar reaction in terms of emotions and concerns at the population level. Our study investigated the impact of the COVID-19 pandemic on psychological well-being in a cohort of Italian university students.

**Methods:**

We conducted a cross-sectional survey in the period immediately after the first lockdown through the administration of a questionnaire on the personal websites of students attending their undergraduate courses at the Università Cattolica del Sacro Cuore. We used the Patient-Health-Engagement-Scale, Self-Rating-Anxiety-Scale, and Self-Rating-Depression-Scale to assess engagement, anxiety symptoms, and depression symptoms of our sample.

**Results:**

The sample size was 501 subjects, of which 35.33% were classified as anxious and 72.93% as depressed. Over 90% of respondents had good understanding of the preventive measures despite over 70% suffered from the impossibility of physically seeing friends and partners. Around 55% of students would have been willing to contribute much more to face the pandemic. An increase in the occurrences of anxiety was associated with being female, being student of the Rome campus, suffering from the impossibility of attending university, being distant from colleagues, and being unable of physically seeing one’s partner. Performing physical activity reduced this likelihood.

**Conclusion:**

University students are at risk of psychological distress in the case of traumatic events. The evolution of the pandemic is uncertain and may have long-term effects on mental health. Therefore, it is crucial to study the most effective interventions to identify vulnerable subgroups and to plan for acute and long-term psychological services to control and reduce the burden of psychological problems.

**Supplementary Information:**

The online version contains supplementary material available at 10.1186/s12992-021-00680-w.

## Introduction

The first outbreak of the novel coronavirus diseases (COVID-19) was reported at the end of December 2019 in Wuhan, China and rapidly the virus spread globally; on March 11, 2020, the World Health Organization declared a pandemic state [1]. The first country affected in Europe was Italy, where the epidemic began on February 21, with Lombardy being the epicenter of COVID-19 cases and deaths (representing 39 and 48% of the total, respectively). As of November 01, 2020, the Italian national surveillance system had reported 309,335 cases and 38,826 deaths from COVID-19 [2], while during the first phase of the pandemic the highest case fatality rate and one of the highest case mortality rate in Europe was reported [3]. To limit the diffusion of the virus, the Italian government established a series of decrees aimed at containing the spread of the epidemic. First, on February 23, 11 municipalities in Northern Italy, including Lombardy [4], were placed on lockdown. Two days later, the measures were extended to six regions, and on March 11, 2020, the lockdown was extended to the whole national territory until May 3, 2020 [5]. During this period, people could leave their homes only for specific needs (work, health emergencies, and food and drug supplies), schools and universities were closed, inter-regional mobility was suspended, and all types of gatherings were prohibited. Inter-regional mobility was allowed after June 3, and for the first time since May 3, 2020, it was for persons, residing in different regions, possible to return to their places of residence. In some regions, trustee home isolation was compulsory when individuals entered the region.

As during past outbreaks, such as those related to Severe Acute Respiratory Syndrome (SARS) in 2003, Influenza A (H1N1) in 2009, and Ebola in 2014 [6, 7], a number of experts across the world anticipated that COVID-19 will affect the population’s health in psychological, social, and neuroscientific dimensions [8]. Indeed, the pandemic led in the general population to a high incidence of mental health disorders, such as acute stress, post-traumatic stress, anxiety, depression, irritability, insomnia, and decreased attention [6, 9], and these symptoms were more common in individuals with epidemic-related experiences [10]. In particular, the COVID-19 pandemic had a huge impact on the mental health of people in many countries around the world causing similar reaction in terms of emotions and concerns at the population level [11, 12]. In fact, an increase in mental health disorders, especially anxiety and depression, in many Asian and European countries — the first continents affected by the pandemic — was demonstrated resulting in an anxiety and depression prevalence of 32.9 and 35.3%, respectively in Asia and a stress prevalence of 31.9% in Europe [12, 13]. Considering Italy, as it was the first European country affected, the COVID-19 pandemic represented a novelty that generated fear, anxiety and depression, especially in the young and elderly population, with a prevalence of anxiety, depression and stress of 18.7, 32.7 and 27.2%, respectively [14]. Indeed, uncertainties related to the viruses characteristics, absence of treatments, rapid spread and lack of protective devices created a huge source of stress resulting in common health disorders [15, 16].

University students are a special social group with active life habits based on relationships and contacts, physical and university activities, travel, and gatherings. The pandemic emergency changed their life drastically:  considering university restrictions, indeed, teaching in presence was suspended from March 11, 2020 until the beginning of September 2020. Only faculty and administrative technical staff were allowed to access the campuses. At the same time, online teaching service had been activated, through which lessons, exams, and theses and doctoral dissertation discussions were carried out at distance. Student attendance was allowed again in September and October, with a combination of face-to-face and distance teaching with the possibility for students to choose which method to use. In case of face-to-face teaching, reservation was necessary. Moreover, the lessons were organized to avoid the presence of different course years to prevent gatherings. As far as the health professions were concerned, traineeships in hospital have been maintained in presence. In this context, the 71 days of total lockdown might have facilitated the development of mental health disorders, especially anxiety and depression [17, 18]. For these reasons, the objective of this study was to evaluate the impact of the COVID-19 outbreak on the well-being in a cohort of university students during the first wave of pandemic and related lockdown.

## Methods

A web-based survey was conducted between June 8 and July 12, 2020, in the period immediately after the lockdown. We administered an anonymous questionnaire of 90 items on the personal websites of students attending undergraduate courses at the Università Cattolica del Sacro Cuore. The university has 4 campuses in 3 Italian regions: Milan (Lombardy), Brescia (Lombardy), Piacenza-Cremona (Lombardy and Emilia-Romagna), and Rome (Lazio). Students belong to the faculties of Medicine and Surgery (Rome), Psychology (Milan and Brescia), Economy (Rome), Economy and Law (Piacenza-Cremona), Agricultural, Food, and Environmental Sciences (Piacenza-Cremona), Banking, Financial, and Insurance Sciences (Milan), and Education Sciences (Milan). Participation was voluntary and unpaid. To participate, students had to give their informed consent. The protocol of the study was approved by the Ethics Committee of the Policlinico Universitario A. Gemelli IRCCS and by the Internal Board of the University.

### Instruments

#### Dependent variables

We used level of anxiety and depression as dependent variables of this study. Students’ level of anxiety was assessed with the Zung Self-Rating Anxiety Scale (SAS), which is composed of 20 items with 4 possible answers (rarely, sometimes, often, most of the time) investigating anxiety levels based on scoring of autonomic, cognitive, motor, and central nervous systems’ symptoms (such as “I feel more nervous and anxious than usual” or “I feel that everything is all right and nothing bad will happen”). In this way, each item is scored on a Likert scale ranging from 1 to 4. A total score < 50 corresponds with absence of anxiety, while 50–59, 60–69, and > 70 indicate slight, moderate, and severe anxiety, respectively [19].

Depression level was explored through the Zung Self-Rating Depression Scale (SDS). The scale comprises 20 items covering affective, psychological, and somatic symptoms associated with depression (such as “I have trouble sleeping at night” or “I am restless and can’t keep still”). In this case, there are also 4 possible answers (rarely, sometimes, often, most of the time), and each item is scored on a Likert scale ranging from 1 to 4. A total score < 50 corresponds to absence of depression, while 50–59, 60–69, and > 70 indicate slight, moderate, and severe depression, respectively [20]. Both scales have been proven reliable and were translated into Italian before being administered to the students in the survey [19, 20].

#### Independent variables

##### General characteristics of students

Students’ demographic information (age, gender, date of birth), faculty, year of study, residence region, presence of a partner, health condition related to COVID-19, family and friends’ health conditions related to COVID-19, and lifestyle during the lockdown (physical activity and whether they lived alone) were collected.

##### Feelings and fears about the pandemic

The validated Patient Health Engagement Scale (PHE-S) evaluated emotions, feelings, concerns, perceptions, and psychological engagement of students. In particular, the scale consists of 5 items with scores of a purely ordinal categorical and psychometric nature describing how people feel when thinking about their own health (such as “I can’t understand what happened to me” or “I feel positive”). Each sentence can be completed by choosing one of 4 specific states or the intermediate points between two states [21]. According to the score obtained, each respondent is determined to have 1 of the 4 levels of health engagement described by the PHE model (i.e., blackout, arousal, adhesion, eudaimonic project). The scale is based on the assumption that the score a person obtains should reflect his or her actual health engagement level. Moreover, 8 questions investigated the fear about an increase of COVID-19 cases, deaths, risk of contagion, capacity to contain the diffusion of the virus, and understanding of preventive measures.

##### Personal concerns regarding university studies

Specific items investigated personal concerns about university studies during the COVID-19 pandemic (impossibility of attending university, concentration, distance from colleagues, and fear returning to university).

### Statistical analysis

The reliability of the scales was measured using Cronbach’s alpha coefficient. The 95% confidence interval (CI) for each alpha value was estimated using 1000 bootstrap samples. Descriptive analyses were performed for all variables. Univariable and multivariable logistic regressions were conducted to assess the influence of independent variables (general characteristics of students, feelings and fears about the pandemic, personal concerns regarding university studies) on each binary outcome (anxiety and depression), with the results expressed as odds ratios (OR), 95% CI. *P*-values below 0.05 were considered statistically significant. All statistical analyses were performed using Stata software, version 14 (StataCorp LP, College Station, TX).

## Results

Five hundred fifty-five questionnaires were collected, of which 501 were used for the analysis, with an effective rate of 90.27%. In all, 54 questionnaires were excluded because they had incomplete information about gender, course of study, or campus. The SAS, SDS and PHE scales showed high reliability rates, with a Cronbach’s alpha coefficient of 0.90 (95% CI [0.88–0.91]), 0.89 [0.87–0.90] and 0.76 [0.72–0.80], respectively.

Table 1 provides a description of the general characteristics of the students. Their median age was 22.9 years (IQR = 21.08–24.56), and females accounted for 71.46% of the total. Around 61% of the respondents were Medicine and Surgery Faculty students of the Rome Campus, with a higher prevalence of first-year students (21.36%). Most students were resident in Northern and Southern Italy (36.13 and 37.92%, respectively), and a large majority reported having returned home during the lockdown (65.67%). Of the students, 71% reported having engaged in physical activity during the lockdown. Overall, 11.78% of the students reported suffering from COVID-19-like symptoms, and, among the 32 who performed a swab test, 6 (18.75%) had a positive result, with 4 asymptomatic in isolation and 2 hospitalized.

**Table 1:** General characteristics of students

Variable	Category	Number	Percent
Age	Median (IQR) = 22.9 (21.08–24.56)		
Gender	Male	143	28.54
	Female	358	71.46
Course of study	Economy	9	1.79
	Economy and Law	37	7.38
	Medicine and Surgery	304	60.68
	Psychology	108	21.56
	Agricultural, Food, and Environmental Sciences	41	8.18
	Banking, Financial, and Insurance Sciences	1	0.2
	Education Sciences	1	0.2
Campus	Brescia	15	2.99
	Milan	94	18.76
	Piacenza-Cremona	80	15.97
	Rome	312	62.28
Year of course	1	107	21.36
	2	95	18.96
	3	79	15.77
	4	68	13.57
	5	60	11.98
	6	77	15.37
	Other	15	2.99
Area of residence	North	181	36.13
	Central	130	25.95
	South and Island	190	37.92
Returned home	No	172	34.33
	Yes	329	65.67
Lived alone during the lockdown	No	455	94.71
	Yes	46	9.18
Physical activity during the lockdown	No	143	28.54
	Yes	358	71.46
Known positive cases	No	329	65.67
	Yes	159	31.74
COVID-19-like symptoms	No	429	85.63
	Yes	59	11.78
	Missing	13	2.59
Swab	No	446	89.02
	Yes	32	6.39
	Missing	23	4.59
Positive swab	No	26	81.25
	Yes	6	18.75
Clinical condition of positive cases	Asymptomatic in isolation	4	66.67
	Hospitalization	2	33.33
Partner	No	234	46.71
	Yes	241	48.1
	Missing	26	5.19
Anxiety (among students with anxiety, % of severity level)	No	302	64.66
	Slight	118	25.26 (71.51)
	Moderate	31	6.63 (18.78)
	Severe	16	3.42 (9.69)
Depression (among students with depression, % of severity level)	No	121	27.06
	Slight	263	58.83 (80.67)
	Moderate	60	13.42 (18.40)
	Severe	3	0.67 (0.92)

According to the SAS, 35.33% of the students (*n* = 165) were classified as anxious and, among students with anxiety, 71.51% had slight anxiety. The SDS classified 72.93% as depressed (*n* = 326) with mainly (80.67%) slight depression (Table 1). Students’ feelings, fears, and PHE scale results related to the effects of COVID-19 on students’ lives are reported in Table S1. Almost 40% of the students referred to have fears about whether the pandemic was under control, and more than 60% referred to fear the increase in positive cases and deaths (64.47 and 68.86%, respectively). Over 90% of the respondents reported understanding the lockdown’s preventive measures despite 70.26% suffered from the impossibility of seeing friends and 75.94% suffered from not seeing their partners. Most (55.69%) students reported being willing to contribute much more to face the pandemic. The PHE-S assessed the engagement level of students related to COVID-19: 16 (3.28%), 100 (20.49%), 317 (64.96%), and 55 (11.27%) students had an engagement level of 1, 2, 3, and 4, respectively.

Table S2 summarizes students’ personal concerns about their studies. More than 60% of the students suffered from the impossibility of attending university and 54.09% being distant from their fellow students. Others (35.03%) reported being concerned about the possibility that the pandemic could reduce their study activities, and 30.14% were concerned that the preventive measures could hinder their studies. About 21% of the respondents stated that they were worried about returning to university, and 39.72% were afraid about their future careers due to the effect of COVID-19 on the country’s economy and on the labor market. Almost one-third (30.14%) declared that they felt optimistic about a solution to the pandemic, and 44.32% declared that they were even more determined to complete their studies (Table S2).

### Predictors of anxiety

Table 2 reports the distributions of the selected covariates and adjusted ORs for anxiety.

**Table 2:** Predictors of anxiety and depression (Adjusted OR: Odds Ratio; CI: 95% confidence interval)

**ANXIETY**					
**Variable**	**Category**	**Anxiety**			
		No	Yes	OR [95% CI]	P-value
		N (%)	N (%)		
Total		302 (64.67)	165 (35.33)		
Gender	Male	104 (77.04)	31 (22.96)	–	
	Female	198 (59.64)	134 (40.36)	2.44 [1.769–3.861]	**< 0.0001**
		Median (IQR)	Median (IQR)		
Age		23.1 (3.49)	22.5(3.52)	0.97 [0.94–1.004]	0.083
Area of residence	North	115 (68.86)	52 (31.14)	–	
	Center	78 (65.0)	42 (35.0)	1.08 [0.517–2.267]	0.883
	South and Island	109 (60.56)	71 (39.44)	1.36 [0.701–2.663]	0.358
Faculty	Other	62 (75.61)	20 (24.39)	–	
	Medicine	176 (61.32)	111 (38.68)	3.62 [0.422–31.155]	0.240
	Psychology	64 (65.31)	34 (34.69)	1.20 [0.605–2.382]	0.600
Campus	(Brescia Milano Piacenza-Cremona)	120 (69.36)	53 (30.64)	–	
	Rome	182 (61.90)	112 (38.10)	1.55 [1.032–2.340]	**0.035**
Live alone	No	276 (65.25)	147 (34.75)	–	
	Yes	26 (59.09)	18 (40.91)	1.29 [0.67–2.48]	0.451
Know people who have tested positive	No	217 (66.77)	108 (33.23)	–	
	Yes	85 (59.86)	57 (40.14)	1.49 [0.97–2.29]	0.068
Engage in physical activities	No	75 (56.82)	57 (43.18)	–	
	Yes	227 (67.76)	108 (32.24)	0.58 [0.383–0.901]	**0.015**
Suffering from the impossibility of seeing one’s partner	Strongly disagree	27 (77.14)	8 (22.86)	–	
	Disagree	2 (66.67)	1 (33.33)	1.34 [1.07–1.67]	**0.010**
	Moderate	15 (88.24)	2 (11.76)		
	Agree	38 (71.70)	15 (28.30)		
	Strongly agree	71 (55.47)	57 (44.53)		
Concerned about COVID-19	Strongly disagree	18 (78.26)	5 (21.74)	–	
	Disagree	79 (68.70)	36 (31.30)	1.27 [1.03–1.56]	**0.023**
	Moderate	116 (65.17)	62 (34.83)		
	Agree	78 (61.90)	48 (38.10)		
	Strongly agree	11 (44.00)	14 (56.00)		
Pandemic feels like something distant	Strongly disagree	198 (61.68)	123 (38.32)	–	
	Disagree	80 (68.97)	36 (31.03)	0.68 [0.492–0.949]	**0.023**
	Moderate	17 (73.91)	6 (26.09)		
	Agree	6 (100.0)	0		
	Strongly agree	1 (100.0)	0		
Understand preventive measures	Strongly disagree	0	0	–	
	Disagree	1 (50)	1 (50.0)	0.79 [0.56–1.11]	0.166
	Moderate	9(60)	6 (40.0)		
	Agree	88 (62.86)	52 (37.14)		
	Strongly agree	204 (65.81)	106 (34.19)		
Fear about containment of the pandemic	Strongly disagree	32 (80.0)	8 (20.0)	–	
	Disagree	80 (78.43)	22 (21.57)	1.66 [1.383–2.007]	**< 0.0001**
	Moderate	106 (72.60)	40 (27.40)		
	Agree	59 (50.86)	57 (49.14)		
	Strongly agree	25 (39.68)	38 (60.32)		
Fear about the increase in positive cases	Strongly disagree	7 (58.33)	5 (41.67)	–	
	Disagree	35 (81.40)	8 (18.60)	1.41 [1.155–1.737]	**0.001**
	Moderate	70 (73.68)	25 (26.32)		
	Agree	118 (69.01)	53 (30.99)		
	Strongly agree	72 (49.32)	74 (50.68)		
Fear about the increase in deaths	Strongly disagree	6 (66.67)	3 (33.33)	–	
	Disagree	30 (85.71)	5 (14.29)	1.60 [1.28–2.00]	**< 0.0001**
	Moderate	65 (77.38)	19 (22.62)		
	Agree	113 (68.90)	51 (31.10)		
	Strongly agree	88 (50.29)	87 (49.71)		
Suffering from distance to fellow students	Strongly disagree	41 (74.55)	14 (25.45)	–	
	Disagree	42 (73.68)	15 (26.32)	1.35 [1.156–1.590]	**< 0.0001**
	Moderate	58 (67.44)	28 (32.56)		
	Agree	110 (70.97)	45 (29.03)		
	Strongly agree	51 (44.74)	63 (55.26)		
Suffering from impossibility of attending university	Strongly disagree	37 (77.08)	11 (22.92)	–	
	Disagree	40 (76.92)	12 (23.08)	1.37 [1.167–1.616]	**< 0.0001**
	Moderate	45 (71.43)	18 (28.57)		
	Agree	101 (64.74)	55 (35.26)		
	Strongly agree	79 (53.38)	69 (46.62)		
Desire to contribute much more to facing the pandemic	Strongly disagree	12 (75.0)	4 (25.0)	–	
	Disagree	33 (75.0)	11 (25.0)	1.33 [1.095–1.636]	**0.004**
	Moderate	90 (69.23)	40 (30.77)		
	Agree	112 (67.88)	53 (32.12)		
	Strongly agree	55 (49.11)	57 (50.89)		
Suffering from the impossibility of playing sports outside	Strongly disagree	88 (65.19)	47 (34.81)	–	
	Disagree	70 (69.31)	31 (30.69)	1.07 [0.93–1.23]	0.317
	Moderate	56 (60.22)	37 (39.78)		
	Agree	49 (70.0)	21 (30.0)		
	Strongly agree	39 (57.35)	29 (42.65)		
Suffering from the impossibility of seeing one’s partner during the lockdown	Strongly disagree	27 (77.14)	8 (22.86)	–	
	Disagree	2 (66.67)	1 (33.33)	1.20 [1.004–1.450]	**0.045**
	Moderate	15 (88.24)	2 (11.76)		
	Agree	38 (71.70)	15 (28.30)		
	Strongly agree	71 (55.47)	57 (44.53)		
Concerned about the possibility that the pandemic could reduce one’s concentration on academic activities	Strongly disagree	69 (88.46)	9 (11.54)	–	
	Disagree	61 (75.31)	20 (24.69)	1.92 [1.61–2.29]	**< 0.0001**
	Moderate	70 (68.63)	32 (31.37)		
	Agree	62 (61.39)	39 (38.61)		
	Strongly agree	20 (26.32)	56 (73.68)		
Concerned that preventive measures could hinder one’s studies	Strongly disagree	79 (81.44)	18 (18.56)	–	
	Disagree	72 (75.0)	24 (25.0)	1.67 [1.42–1.96]	**< 0.0001**
	Moderate	60 (63.83)	34 (36.17)		
	Agree	52 (56.52)	40 (43.48)		
	Strongly agree	19 (32.20)	40 (67.80)		
Concerned about returning to university	Strongly disagree	111 (70.25)	47 (29.75)	–	
	Disagree	75 (72.82)	28 (27.18)	1.25 [1.08–1.44]	**0.003**
	Moderate	41 (56.16)	32 (43.84)		
	Agree	28 (59.57)	19 (40.43)		
	Strongly agree	27 (47.37)	30 (52.63)		
Concerned about future career because of the COVID-19 pandemic	Strongly disagree	63 (73.26)	23 (26.74)	–	
	Disagree	47 (63.51)	27 (36.49)	1.26 [1.09–1.46]	**0.002**
	Moderate	51 (64.56)	28 (35.44)		
	Agree	78 (70.27)	33 (29.73)		
	Strongly agree	43 (48.86)	45 (51.14)		
Determined to complete studies	Strongly disagree	16 (48.48)	17 (51.52)	–	
	Disagree	34 (60.71)	22 (39.29)	0.81 [0.69–0.96]	**0.014**
	Moderate	80 (62.99)	47 (37.01)		
	Agree	73 (65.18)	39 (34.82)		
	Strongly agree	79 (71.82)	31 (28.18)		
Feel optimistic about a solution to the pandemic	Strongly Disagree	20 (40.82)	29 (59.18)		
	Disagree	58 (59.79)	39 (40.21)	0.71 [0.59–0.85]	**< 0.0001**
	Moderate	93 (65.96)	48 (34.04)		
	Agree	86 (74.14)	30 (25.86)		
	Strongly agree	25 (71.43)	10 (28.57)		
Patient health engagement scale	4	45 (85.33)	9 (16.67)	–	
	3	213 (70.07)	91 (29.93)	2.71 [1.923–3.846]	**< 0.0001**
	2	42 (44.68)	52 (55.32)		
	1	2 (13.33)	13 (86.67)		
**DEPRESSION**					
**Variable**	**Category**	**Depression**			
		No	Yes	OR [95% CI]	*p* value
		N (%)	N (%)		
Total		121 (27.07)	326 (72.93)		
Gender	Male	38	93	–	
	Female	83	233	1.15 [0.73–1.82]	0.553
		Median (IQR)	Median (IQR)		
Age		23.18 (3.67)	22.71 (3.39)	0.994 [0.957–1.031]	0.743
Area of residence	North	48 (30.38)	110 (69.62)	–	
	Center	25 (21.19)	93 (78.81)	1.21 [0.555–2.678]	0.621
	South and Island	48 (28.07)	123 (71.93)	0.87 [0.438–1.730]	0.695
Faculty	Other	28 (36.84)	48 (63.16)	–	
	Medicine	68 (24.46)	210 (75.54)	0.60 [0.694–5.291]	0.651
	Psychology	25 (26.88)	68 (73.12)	1.64 [0.825–.265]	0.157
Campus	(Brescia Milano Piacenza-Cremona)	52 (31.90)	111 (68.10)	–	
	Rome	69 (24.30)	215 (75.70)	1.49 [0.970–.297]	**0.068**
Lived alone during the lockdown	No	109 (26.85)	297 (73.15)	–	
	Yes	12 (29.27)	29 (70.73)	0.82 [0.401–1.685]	0.740
Known positive cases	No	86 (27.65)	225 (72.35)	–	
	Yes	35 (25.74)	101 (74.26)	1.209 [0.754–1.937]	0.431
Physical activity during the lockdown	No	42 (33.33)	84 (66.67)	–	
	Yes	79 (24.61)	242 (75.39)	1.57 [0.996–2.488]	0.100
Suffering from the impossibility of seeing one’s partner during the lockdown	Strongly disagree	8 (23.53)	26 (76.47)	–	
	Disagree	1 (33.33)	2 (66.67)	1.12 [0.937–1.354]	0.2045
	Moderate	8 (23.53)	9 (52.94)		
	Agree	16 (30.77)	36 (69.23)		
	Strongly agree	28 (22.95)	94 (77.059		
Concerned about COVID-19	Strongly disagree	4 (19.05)	17 (80.95)	–	
	Disagree	31 (28.44)	78 (71.56)	1.01 [0.813–1.259]	0.917
	Moderate	48 (28.07)	123 (71.93)		
	Agree	34 (27.87)	88 (72.13)		
	Strongly agree	4 (16.67)	20 (82.33)		
Pandemic feels like something distant	Strongly disagree	81 (26.05)	230 (73.95)	–	
	Disagree	31 (28.44)	78 (71.56)	0.94 [0.690–1.289]	0.715
	Moderate	9 (45.0)	11 (55.0)		
	Agree	0	6 (100.0)		
	Strongly agree	0	1 (100.0)		
Understand preventive measures	Strongly disagree	0	0	–	
	Disagree	1 (50.0)	1 (50.0)	1.19 [0.83–.69]	0.331
	Moderate	7 (46.67)	8 (53.33)		
	Agree	34 (25.56)	99 (74.44)		
	Strongly agree	79 (26.60)	218 (73.40)		
Fear about containment of the pandemic	Strongly disagree	6 (15.79)	32 (84.21)	–	
	Disagree	32 (32.99)	65 (67.01)	0.97 [0.802–1.166]	0.731
	Moderate	37 (26.62)	102 (73.38)		
	Agree	32 (28.57)	80 (71.43)		
	Strongly agree	14 (22.95)	47 (77.05)		
Fear about the increase in positive cases	Strongly disagree	3 (25.0)	9 (75.0)	–	
	Disagree	12 (27.91)	31 (72.09)	0.89 [0.724–1.098]	0.283
	Moderate	18 (19.57)	74 (80.43)		
	Agree	49 (30.25)	113 (69.75)		
	Strongly agree	39 (28.26)	99 (71.74)		
Fear about the increase in deaths	Strongly disagree	2 (22.22)	7 (77.78)	–	
	Disagree	7 (20.0)	28 (80.0)	0.80 [0.64–1.10]	0.101
	Moderate	17 (20.99)	64 (79.01)		
	Agree	45 (28.85)	45 (28.85)		
	Strongly agree	50 (30.12)	50 (30.12)		
Suffering from the distance from one’s fellow students	Strongly disagree	17 (33.33)	34 (66.67)	–	
	Disagree	21 (37.50)	35 (62.50)	1.15 [0.993–1.369]	**0.060**
	Moderate	18 (22.22)	63 (77.78)		
	Agree	42 (27.81)	109 (72.19)		
	Strongly agree	23 (21.30)	85 (78.70)		
Suffering from the impossibility of attending university	Strongly disagree	14 (30.43)	32 (69.57)	–	
	Disagree	17 (34.69)	32 (65.31)	1.12 [0.957–1.313]	0.155
	Moderate	14 (24.14)	55 (75.86)		
	Agree	44 (28.95)	108 (71.05)		
	Strongly agree	32 (22.54)	110 (77.46)		
Desire to contribute much more to facing the pandemic	Strongly disagree	4 (25.0)	12 (75)	–	
	Disagree	12 (27.27)	32 (72.73)	1.12 [0.921–1.379]	0.243
	Moderate	40 (32.26)	84 (67.74)		
	Agree	46 (29.68)	109 (70.32)		
	Strongly agree	19 (17.59)	89 (82.41)		
Suffering from the impossibility of playing sports outside	Strongly disagree	35 (27.13)	94 (72.87)	–	
	Disagree	31 (32.98)	63 (67.02)	1.06 [0.91–1.23]	0.424
	Moderate	22 (24.44)	68 (75.56)		
	Agree	17 (24.64)	52 (75.36)		
	Strongly agree	16 (24.62)	49 (75.38)		
Suffering from the impossibility of seeing one’s partner during the lockdown	Strongly Disagree	8 (25.53)	26 (76.47)	–	
	Disagree	1 (33.33)	2 (66.67)	1.12 [0.937–1.354]	0.205
	Moderate	8 (47.06)	9 (52.94)		
	Agree	16 (30.77)	36 (69.23)		
	Strongly Agree	28 (22.95)	94 (77.05)		
Concerned about the possibility that the pandemic could reduce one’s concentration on academic activities	Strongly Disagree	17 (21.79)	61 (78.21)	–	
	Disagree	22 (27.16)	59 (72.84)	0.90 [0.77–1.06]	0.201
	Moderate	30 (29.41)	72 (70.59)		
	Agree	27 (26.73)	74 (73.27)		
	Strongly Agree	23 (30.26)	53 (69.74)		
Concerned that preventive measures could hinder one’s studies	Strongly Disagree	19 (19.59)	78 (80.41)	–	
	Disagree	23 (23.96)	73 (76.04)	0.88 [0.75–1.03]	0.115
	Moderate	35 (37.23)	59 (62.77)		
	Agree	27 (29.35)	65 (70.65)		
	Strongly Agree	15 (25.42)	44 (74.58)		
Concerned about returning to university	Strongly Disagree	34 (21.52)	124 (78.48)	–	
	Disagree	33 (32.04)	70 (67.96)	0.93 [0.80–1.09]	0.374
	Moderate	24 (32.88)	49 (67.12)		
	Agree	15 (31.91)	32 (68.09)		
	Strongly Agree	13 (22.81)	44 (77.19)		
Concerned about future career because of the COVID-19 pandemic	Strongly Disagree	19 (22.09)	67 (77.91)	–	
	Disagree	22 (29.73)	52 (70.27)	0.99 [0.85–1.16]	0.956
	Moderate	21 (26.58)	58 (73.42)		
	Agree	37 (33.33)	74 (66.67)		
	Strongly Agree	20 (22.73)	68 (77.27)		
Determined to complete studies	Strongly Disagree	7 (21.21)	26 (78.79)	–	
	Disagree	20 (35.71)	36 (64.29)	1.07 [0.89–1.27]	0.475
	Moderate	37 (29.13)	90 (70.87)		
	Agree	27 (24.11)	85 (75.89)		
	Strongly Agree	28 (25.45)	82 (74.55)		
Feeling optimistic about a solution to the pandemic	Strongly Disagree	13 (26.53)	36 (73.47)	–	
	Disagree	30 (30.93)	67 (69.07)	1.12 [0.93–1.36]	0.242
	Moderate	40 (28.37)	101 (71.63)		
	Agree	30 (25.86)	86 (74.14)		
	Strongly Agree	6 (17.14)	29 (82.86)		
Patient health engagement scale	4	8 (15.69)	43 (84.31)	–	
	3	88 (30.03)	205 (69.97)	0.97 [0.69–1.33]	0.828
	2	24 (26.97)	65 (73.03)		
	1	1 (7.14)	13 (92.86)		

Being female was a risk factor, while performing physical activity was a protective factor (Adjusted Odds ratio: OR 2.44, 95% CI [1.769–3.861] and OR 0.58, 95% CI [0.383–0.901], respectively). Students of the Rome campus were more likely to have anxiety compared to those of other campuses (OR 1.55, 95% CI [1.032–2.340]).

Concern about COVID-19, fear about the containment of the pandemic and about the increase in positive cases and deaths were risk factors for the occurrence of anxiety (OR 1.27, 95% CI [1.03–1.56]; OR 1.41, 95% CI [1.155–1.737]; OR 1.60, 95% CI [1.28–2.00]). Similarly, suffering from the impossibility attending university, distance from fellow students, and impossibility seeing partners was associated with increased occurrence of anxiety (OR 1.37, 95% CI [1.167–1.616]; OR 1.35, 95% CI [1.156–1.590]; OR 1.34, 95% CI [1.07–1.67]). Additionally, the probability of having anxiety was higher in students worried about the possibility that the pandemic could reduce study activities, about returning to university, and about their future careers due to the COVID-19 pandemic (OR 1.92, 95% CI [1.61–2.29]; OR 1.25, 95% CI [1.08–1.44]; OR 1.26, 95% CI [1.09–1.46]).

### Predictors of depression

Table 2 presents the distributions of the selected covariates and adjusted ORs for depression. Students of the Rome Campus were more likely to experience depression compared to those of the other campuses, even if the association was borderline (OR 1.49, 95% CI [0.970–2.297]). The distance from one’s fellow students was a borderline risk factor for the occurrence of depression (OR 1.15 95% CI [0.993–1.369]). No significant association was found with the other variables.

## Discussion

Our results show that 35.33% of our sample of university students had symptoms of anxiety and 72.93% of depression, although with mild symptoms. These data are in line with previous studies that demonstrated how young people, in particular university students, are at greater risk for psychological distress in case of a health emergency [22]. This confirms that the pandemic increased common mental health disorders across the population, with a prevalence of anxiety and depression of about 32.9 and 35.3% in Asia and 23.8 and 32.4% in Europe, respectively [12]. Specifically, in Italy, rates of anxiety and depression in the general population are in line with these finding (18.7–20.8% and 17.3–32.7%, respectively) [14, 15]. However, our student cohort shows a prevalence of anxiety and depression almost twice as high as that observed in the general population. This finding is in line with what has been observed in other studies conducted in European countries such as the United Kingdom, France, and Greece [23–25], where an increase in anxiety and depression was observed during the pandemic and particularly during the lockdown period in university students. Among non-European Union countries, analyzing student populations, a Chinese study observed slightly lower values of anxiety (about 25%) [17], although these values were still higher than those found in the general population [26]. Therefore, students represent a vulnerable population for common mental health disorders. In particular, unsettled life and work conditions, typical of academic environments, and the life stage of university students, who are “in transition” to adulthood and in a delicate process of starting their careers, makes them more susceptible to negative psychological effect of traumatic events [27]. In line with this interpretation, the psychological distress levels measured in our study are associated with students’ concerns about their academic activities, both in terms of delays regarding completion of their degrees [17] and their sense of loneliness and isolation due to physical distance from their peers and partners [28, 29] in relation with COVID-19 effects and its containment measures.

Furthermore, our study showed that students attending the Rome campus presented higher levels of anxiety. This may be due to the prominence of medical students at this campus, who are at higher risk of psychological distress due to their familiarity with health issues compared to the general population [17, 30, 31]. Moreover, this result may be due to the fact that individuals involved in medical professions are typically more empathetic and altruistic and tend to be at higher risk for negative psychological reactions in an health crisis situation [32]. This evidence is also in line with previous studies that showed higher psychological distress in individuals with geographical proximity to the regions mainly affected by the pandemic (in our case, the North of Italy). The sense of lacking control over the current situation (due to geographical distance from the “red regions” for the Italian COVID-19 epidemic) and the emotional appraisal of the situation from individuals living in Rome, based mainly on the accounts of other individuals more directly affected by the emergency or on the media coverage of the emergency, seem to have emphasized the negative psychological effects of the health crisis in a sort of “psychological eco process” [33]. Furthermore, our data show that, increased willingness of students to contribute in efforts controlling the pandemic increased levels of anxiety, thus confirming that the feeling of losing control over one’s own health risk management can trigger psychological distress [14]. The study demonstrated a relationship between gender and anxiety level confirming previous studies in which females tended to develop more anxiety symptoms in reaction to health emergencies and imposed quarantine than their male counterparts [34]. This relationship is controversial since other studies reported higher anxiety scores in males [26]. This difference may be the result of cultural factors shaping gender-related attitudes and behaviors.

Finally, the level of physical activity performed during the quarantine resulted in a protective factor against psychological distress. The beneficial effect of physical activity on mental well-being has been widely shown in literature [35, 36]. Furthermore, recent studies demonstrated that exercising and physical activity during quarantine is critical to promote both mental and physical health [36–38], particularly for younger people.

To our knowledge, this is the first study to investigate the impact of the COVID-19 pandemic on the mental health and psychological well-being of Italian university students during the first wave. Moreover, the sample was representative of Italian students because the Università Cattolica del Sacro Cuore has campuses in different Italian regions with distinct faculties. Limitations of our analysis are related to its opportunistic sampling and cross-sectional design, which prevents causal interpretations. Another limitation was related to reliance on self-reported measures rather than clinical diagnoses of anxiety and depression, although the selected SAS and SDS scales have been validated and are commonly used. Additionally, considering the web-based distribution, no data were collected regarding non-participating students.

## Conclusion

Our study showed that students are at risk of psychological distress in case of traumatic events, such as health emergencies. Since the evolution of the pandemic is uncertain and effects on mental health may be long-term, it is crucial to study the most effective interventions at school level, identifying the most vulnerable subgroups and planning for acute and long-term psychological services to control and reduce fear, and consequently burden of psychological problems.

In this context, our university has activated an assistance service available for students to assist them in case of problems related to study and teaching activities during the pandemic, as well as a help desk with possibilities for psychological assistance, which can be contacted anonymously by all students. In this way, a concrete tool was offered to assist most fragile students affected by anxiety and depressive symptoms. Considering the didactic activities, we should consider that university life is based on relationships, exchange of opinions and “physical” confrontation. Moreover, our students show how anxiety and depression are greatly related to distance from the university environment, the impossibility of attending the university and confronting themselves with their colleagues. On the other hand, the pandemic has taught us that the possibility using digital tools to ensure teaching and functioning of universities is of fundamental importance [39]. In fact, owing to these systems, it has been possible to continue university activities, without slowing down study and learning process of students. As far as our experience is concerned, students accepted digital modes offered resulting in a wide participation in the lessons and no difficulties related to the way the exams are carried out. Therefore, the experience of online teaching can be considered more than positive. In this context, it could be useful to hypothesize a “blended” teaching system, especially regarding the possibility of collaborations between different universities in different cities or countries, to ensure “digital” learning, which can increasingly expand the knowledge of students, alongside physical presence, which is essential to allow students to appreciate all features, qualities and also difficulties of university life.

## Supplementary Information

**Additional file 1 Table S1.** Feelings and fears about the pandemic and PHE-Scale. **Table S2.** Personal concerns regarding university studies.

## Data Availability

Data were collected and analyzed by the Università Cattolica del Sacro Cuore.
