# Supplementary material for: Impact of the COVID-19 pandemic on psychological well-being of students in an Italian university: a web-based cross-sectional survey
Source: Global Health. 2021 Apr 6;17:39. doi: 10.1186/s12992-021-00680-w (PMC8022300; doi:10.1186/s12992-021-00680-w)
Supplement: Supplementary file 1 — Additional file 1 Table S1. Feelings and fears about the pandemic and PHE-Scale. Table S2. Personal concerns regarding university studies. [file 12992_2021_680_MOESM1_ESM.docx]

**Table S1.** **Feelings and fears about the pandemic and PHE-Scale**

| **Items** | **Category** | **N** | **%** |
| --- | --- | --- | --- |
| Pandemic feels like something distant | Strongly disagree | 323 | 64.47 |
|  | Disagree | 122 | 24.35 |
|  | Moderate | 23 | 4.59 |
|  | Agree | 6 | 1.2 |
|  | Strongly agree | 1 | 0.2 |
|  | Missing | 26 | 5.19 |
| Feel at risk of being contagious | Strongly disagree | 23 | 4.59 |
|  | Disagree | 117 | 23.35 |
|  | Moderate | 181 | 36.13 |
|  | Agree | 128 | 25.55 |
|  | Strongly agree | 26 | 5.19 |
|  | Missing | 26 | 5.19 |
| Fear about containment of the pandemic | Strongly disagree | 40 | 7.98 |
|  | Disagree | 103 | 20.56 |
|  | Moderate | 152 | 30.34 |
|  | Agree | 116 | 23.15 |
|  | Strongly agree | 64 | 12.77 |
|  | Missing | 26 | 5.19 |
| Fear about the increase in positive cases | Strongly disagree | 12 | 2.4 |
|  | Disagree | 43 | 8.58 |
|  | Moderate | 97 | 19.36 |
|  | Agree | 176 | 35.13 |
|  | Strongly agree | 147 | 29.34 |
|  | Missing | 26 | 5.19 |
| Fear about the increase in deaths | Strongly disagree | 9 | 1.0 |
|  | Disagree | 36 | 7.19 |
|  | Moderate | 85 | 16.97 |
|  | Agree | 169 | 33.73 |
|  | Strongly agree | 176 | 35.13 |
|  | Missing | 26 | 5.19 |
| Understand preventive measures | Strongly disagree | 0 | 0 |
|  | Disagree | 3 | 0.6 |
|  | Moderate | 15 | 2.99 |
|  | Agree | 142 | 28.34 |
|  | Strongly agree | 315 | 62,87 |
|  | Missing | 26 | 5.19 |
| Suffering from the impossibility of playing sports outside during the lockdown | Strongly disagree | 135 | 26.95 |
|  | Disagree | 103 | 20.56 |
|  | Moderate | 96 | 19.16 |
|  | Agree | 73 | 14.57 |
|  | Strongly agree | 68 | 13.57 |
|  | Missing | 26 | 5.19 |
| Suffering from the impossibility of seeing friends during the lockdown | Strongly disagree | 18 | 3.59 |
|  | Disagree | 34 | 6.79 |
|  | Moderate | 71 | 14.17 |
|  | Agree | 136 | 27.15 |
|  | Strongly agree | 216 | 43.11 |
|  | Missing | 26 | 5.19 |
| Suffering from the impossibility of seeing one’s partner during the lockdown | Strongly disagree | 36 | 14.94 |
|  | Disagree | 3 | 1.24 |
|  | Moderate | 18 | 7.47 |
|  | Agree | 54 | 22.41 |
|  | Strongly agree | 129 | 53.53 |
|  | Missing | 1 | 0.41 |
| Saw partner after the lockdown | No | 25 | 10.37 |
|  | Yes | 215 | 89.21 |
|  | Missing | 1 | 0.41 |
| Fear of being contagious to partner | No | 107 | 44.4 |
|  | Yes | 133 | 55.19 |
|  | Missing | 1 | 0.41 |
| Fear that partner could be source of contagious | No | 126 | 52.28 |
|  | Yes | 114 | 47.3 |
|  | Missing | 1 | 0.41 |
| Desire to contribute much more to facing the pandemic | Strongly disagree | 16 | 3.19 |
|  | Disagree | 47 | 9.38 |
|  | Moderate | 133 | 26.55 |
|  | Agree | 167 | 33.33 |
|  | Strongly agree | 112 | 22.36 |
|  | Missing | 26 | 5.19 |
| Patient health engagement scale | 1 | 16 | 3.28 |
|  | 2 | 100 | 20.49 |
|  | 3 | 317 | 64.96 |
|  | 4 | 55 | 11.27 |

**Table S2. Personal concerns regarding university studies**

| **Items** | **Category** | **N** | **%** |
| --- | --- | --- | --- |
| Suffering from the impossibility of attending university | Strongly disagree | 49 | 9.78 |
|  | Disagree | 52 | 10.38 |
|  | Moderate | 65 | 12.97 |
|  | Agree | 159 | 31.74 |
|  | Strongly agree | 150 | 29.94 |
|  | Missing | 26 | 5.19 |
| Suffering from the distance from fellow students | Strongly disagree | 56 | 11.18 |
|  | Disagree | 58 | 11.58 |
|  | Moderate | 90 | 17.96 |
|  | Agree | 157 | 31.34 |
|  | Strongly agree | 114 | 22.75 |
|  | Missing | 26 | 5.19 |
| Concerned about the possibility that the pandemic could reduce one’s concentration on academic activities | Strongly disagree | 78 | 15.57 |
|  | Disagree | 81 | 16.17 |
|  | Moderate | 192 | 20.36 |
|  | Agree | 191 | 20.16 |
|  | Strongly agree | 76 | 15.17 |
|  | Missing | 63 | 12.57 |
| Concerned that preventive measures could hinder one’s studies | Strongly disagree | 97 | 19.36 |
|  | Disagree | 96 | 19.16 |
|  | Moderate | 94 | 18.76 |
|  | Agree | 92 | 18.36 |
|  | Strongly agree | 59 | 11.78 |
|  | Missing | 63 | 12.57 |
| Concerned about returning to university | Strongly disagree | 158 | 31.54 |
|  | Disagree | 103 | 20.56 |
|  | Moderate | 73 | 14.57 |
|  | Agree | 47 | 9.38 |
|  | Strongly agree | 57 | 11.38 |
|  | Missing | 63 | 12.57 |
| Concerned about future career because of the COVID-19 pandemic | Strongly disagree | 86 | 17.17 |
|  | Disagree | 74 | 14.77 |
|  | Moderate | 79 | 15.77 |
|  | Agree | 111 | 22.16 |
|  | Strongly agree | 88 | 17.56 |
|  | Missing | 63 | 12.57 |
| Feel optimistic about a solution to the pandemic | Strongly disagree | 49 | 9.78 |
|  | Disagree | 97 | 19.36 |
|  | Moderate | 141 | 28.14 |
|  | Agree | 116 | 23.15 |
|  | Strongly agree | 35 | 6.99 |
|  | Missing | 63 | 12.57 |
| Determined to complete studies | Strongly disagree | 33 | 6.59 |
|  | Disagree | 56 | 11.18 |
|  | Moderate | 127 | 25.35 |
|  | Agree | 112 | 22.36 |
|  | Strongly agree | 110 | 21.96 |
|  | Missing | 63 | 12.57 |
